# Tourist satisfaction as a mediator between event quality and economic outcomes: evidence from a Silk Road heritage destination

**DOI:** 10.3389/fspor.2026.1805388

**Published:** 2026-05-20

**Authors:** Nilufar Omonova, Abror Juraev, Nodira Makhmudova, Gulmira Isokova, Sharifa Hamroyeva, Musharraf Shodieva, Istamkhuja Olimovich Davronov, Shafoat Kadirova, Erkin Farmanov

**Affiliations:** 1 Bukhara State University, Bukhara, Uzbekistan; 2 Bukhara State Medical Institute, Bukhara, Uzbekistan; 3 Russian State Tourism and Service University, Moscow, Russia; 4 Hungarian University of Agriculture and Life Sciences, Gödöllő, Hungary

**Keywords:** destination attractiveness, event tourism, heritage tourism, mediation analysis, structural equation modeling, tourist satisfaction, Uzbekistan

## Abstract

Several studies on event tourism have examined the impact of events on destination attractiveness and economic development. However, relatively little is known about the mechanisms through which event quality influences economic outcomes, particularly in destinations with cultural heritage. Therefore, this study aimed to examine the mediating role of tourist satisfaction in the relationship between event quality and economic outcomes in the Bukhara region, a UNESCO World Heritage site located along the historic Silk Road. A cross-sectional study was conducted with 519 respondents, including international tourists (*n* = 309) and local residents (*n* = 210). Structural equation modeling (SEM) was used to test the hypothesized relationships, and mediation analysis was conducted according to Baron and Kenny's methodology. The results showed that tourist satisfaction mediated 94.1% of the relationship between event quality and economic outcomes, confirming that satisfaction is the predominant mechanism linking event quality to destination economic outcomes. Cultural events demonstrated the greatest effectiveness among event categories, while information availability emerged as a critical gap requiring intervention. Our findings suggest that destinations with cultural heritage should prioritize high-quality event strategies that align with their cultural identity over a proliferation of events. These findings can help destination managers and policymakers optimize event portfolio strategies, promoting both tourist satisfaction and sustainable destination development.

## Introduction

Event tourism has become a strategic tool for developing destinations and enhancing their competitiveness worldwide. Events can create a positive image for a destination and contribute to its geographic and economic development (1). According to the World Tourism Organization, international tourist arrivals reached 1.3 billion in 2023, with events and festivals constituting a significant component of global tourism revenue (2). The sector continues to demonstrate robust growth driven by growing consumer demand for educational travel and cultural interaction. Within this expanding sector, the festival segment leads by type, with a share of approximately 32.50%, underscoring its role as a key driver of event tourism.

The relationship between events and destination attractiveness has received significant academic attention. In their research, Getz and Page found that events function as attractions, image builders, entertainers, and catalysts for destination development. Tourism events that draw on local culture, traditions, and resources have additional appeal and value due to their authenticity, as they can provide participants with a unique and memorable experience (3). Li et al. (4) developed the concept of festival attractiveness. They demonstrated its significant impact on destination loyalty using a mixed-methods study (4), while Rossetti et al. (5) expanded the understanding of the contribution of food festivals to visitor well-being (5). Collectively, these studies highlight the multidimensional benefits that well-designed events provide to host destinations.

Tourist satisfaction has been identified as a central factor in understanding the performance and sustainability of tourism destinations (6). It predicts future behavior, such as repeat visits and referrals, creating a competitive advantage and long-term success. Structural equation modeling (SEM) has become the preferred methodology for studying the complex relationships between event quality, satisfaction, and behavioral intentions (7, 8). Recent applications of SEM in the tourism context have shown that satisfaction acts not simply as an outcome variable, but as a critical mediating mechanism linking service quality to tourist economic behavior (42). For example, Hussain et al. (43) used SEM to examine how tourism satisfaction and service quality influence destination loyalty in a resort setting, confirming the key mediating role of satisfaction (43). Similarly, Wang et al. (9) used moderated mediation analysis to show that tourist satisfaction significantly mediates the relationship between travel experiences and revisit intentions (9). In the context of cultural heritage tourism, Rasoolimanesh et al. (8) applied PLS-SEM and fsQCA to examine visitor satisfaction as a mediator between memorable tourist experiences and behavioral intentions, finding that satisfaction significantly mediates the influence of local culture and involvement on tourists' future behavior. Furthermore, Manojlovic et al. (10) confirmed using SEM that satisfaction derived from cultural tourism serves as a significant predictor of destination loyalty (10). Despite these advances, the exact magnitude of the mediating effect of satisfaction and its implications for event tourism strategies in developing cultural heritage destinations remain understudied.

Tourism destinations present unique opportunities and challenges for event tourism development. Silk Road cities in Central Asia are attracting increasing attention from scholars and specialists; for example, Juraturgunov studied the factors determining loyalty to World Heritage sites within Silk Road tourism in Uzbekistan (11). The number of international tourists visiting Uzbekistan has grown significantly, increasing from 1.9 million in 2014 to 8.2 million in 2024. This dramatic increase reflects government reforms, including visa liberalization for over 90 countries, and significant investments in tourism infrastructure (12).

Despite these changes, significant research gaps remain. First, although the tourism literature recognizes the importance of event quality and tourist satisfaction in shaping behavioral intentions and tourist economic behavior, quantitative data on these mediating pathways remain limited, particularly for emerging cultural heritage sites. Secondly, despite the region's significant cultural assets, there is insufficient research into the criteria for selecting destinations for event tourism in Central Asia. Thirdly, existing assessments of the economic impact of event tourism primarily rely on direct measurement methods, without considering the experience mechanisms – in particular, tourist satisfaction – through which events generate economic value.

The Bukhara region provides an ideal context for addressing these issues. As a UNESCO World Heritage site with 982 registered cultural and historical sites and a recent government priority for event tourism development, Bukhara offers an empirical setting for examining how event quality influences tourist economic behavior (13). The region has seen a significant increase in the number of events: from 18 in 2020 to 112 in 2025, accompanied by a substantial increase in the number of participants and tourism revenues (14).

This study aims to address these gaps by examining the mechanisms through which event tourism influences destination attractiveness in the Bukhara region of Uzbekistan. Specifically, we investigate the following research questions: (1) assess the relationship between event quality aspects and tourist satisfaction; (2) examine whether tourist satisfaction mediates the relationship between event quality and tourist economic behavior; (3) assess the relative effectiveness of different event categories for cultural heritage site development; and (4) identify priority areas for improving event tourism performance based on empirical data.

## Theoretical background

This study uses two complementary theoretical frameworks: expectancy confirmation theory (ECT) and service quality theory to explain the relationship between event quality, tourist satisfaction and economic outcomes.

### Expectancy confirmation theory

Originally proposed by Oliver (15), expectancy confirmation theory (ECT) provides a fundamental framework for understanding how pre-consumption expectations, post-consumption perceptions (confirmation), and resulting satisfaction jointly shape consumers' behavioral intentions (15). In the tourism context, ECT offers a valuable framework for understanding how tourists form loyalty based on their pre-trip motivations and post-trip evaluations (16). The theory postulates that satisfaction arises from the match between pre-visit expectations and post-visit experiences; when actual outcomes exceed expectations, a positive discrepancy occurs, leading to greater satisfaction (9). ECT has been widely applied in tourism research to analyze tourists' satisfaction, loyalty, and behavioral intentions (7). Petrick (17) highlighted how tourists' post-trip satisfaction influences their loyalty and intentions to recommend services, providing an important theoretical foundation for understanding the relationship between satisfaction and loyalty in the context of tourism destinations (17).

### Service quality theory

The service quality paradigm developed within SERVQUAL conceptualizes quality as the difference between customers' expectations and their perceptions of the service provided (18). Although the original SERVQUAL dimensions (tangibles, reliability, responsiveness, assurance, and empathy) have been widely applied in hospitality and tourism research, scholars acknowledged that these dimensions require adaptation to the context of festivals and events (19). The quality of themed festivals is more appropriately measured using attributes such as hospitality, venue, program content, amenities, and overall ambiance (20, 21). Cronin and Taylor (22) provided theoretical rationale and empirical evidence to support the notion that service quality is a predictor of satisfaction, with the quality-satisfaction relationship being validated in a variety of tourism contexts, including sporting events (23), cultural attractions (24), and festivals (20).

#### Event quality and tourist satisfaction

The relationship between service quality and customer satisfaction has been extensively studied in the tourism literature, and findings consistently support a positive correlation (18, 20). Quality has been identified as a direct antecedent of satisfaction in various service contexts (17, 21). Specifically, in the festival and event industry, Armbrecht demonstrated that perceived quality, including both affective and cognitive aspects of event experiences, significantly influences satisfaction (44). A recent study by Hussain used structural modeling to confirm that service quality aspects positively influence tourist satisfaction in the context of tourism destinations (43). Cole and Illum found that service quality significantly influences festival-goer satisfaction (25), while Jeong found that event quality positively influences tourist satisfaction in the context of sports tourism (26). Building on this theoretical and empirical framework, we propose the following hypothesis:

*H1: Event quality has a positive influence on tourist satisfaction.*

#### Tourist satisfaction and behavioral intentions

The relationship between satisfaction and behavioral intentions is one of the most consistent findings in the tourism literature (7, 15). Meta-analytic evidence indicates that satisfaction elicits positive emotions and promotes repeat-visitation behavior, with satisfaction identified as a strong predictor of tourist loyalty. Satisfied tourists are more likely to spread positive word-of-mouth and become repeat visitors (20). Rasoolimanesh applied PLS-SEM to the cultural tourism context and found that satisfaction significantly mediates the influence of memorable experiences on tourists' future behavior, including revisit intentions and recommendations. Manojlovic confirmed using SEM that satisfaction with cultural tourism experiences is a significant predictor of destination loyalty and behavioral intentions. Wang further demonstrated that tourist satisfaction has a significant impact on revisit intentions through a moderation analysis of mediation effects. Based on these results, we hypothesize:

*H2: Tourist satisfaction has a positive impact on behavioral intentions.*

#### Tourist satisfaction and economic outcomes

In addition to behavioral intentions, tourist satisfaction is linked to tangible economic outcomes, including consumption behavior and willingness to pay. Homburg et al. (27) found that satisfied customers exhibit higher willingness to pay. In the tourism context, satisfaction translates into economic value through increased spending on accommodations, dining, shopping, and entertainment. Research on cultural heritage sites shows that tourist satisfaction mediates the relationship between destination characteristics and economic benefits for local communities, with satisfied tourists contributing significantly to local spending (6). The economic consequences of satisfaction are particularly relevant for cultural heritage sites, where visitor spending contributes to both economic prosperity and cultural preservation (28). Therefore, we propose:

*H3: Tourist satisfaction has a positive impact on economic outcomes.*

#### The mediating role of tourist satisfaction

The conceptualization of satisfaction as a mediator between quality perceptions and outcomes has strong theoretical and empirical support. Satisfaction theory explicitly posits satisfaction as an evaluative state that transforms perceptions of quality into behavioral responses (15). In tourism research, numerous studies have confirmed the mediating function of satisfaction. In the context of events, in particular, Cole and Illum (25) demonstrated that the effect of service quality on behavioral intentions (positive reviews and revisit intentions) is fully mediated by festival-goer satisfaction. Jeong et al. (26) further confirmed that tourist satisfaction partially mediates the relationship between event quality and behavioral intentions in sport tourism. Research on cultural heritage sites confirms that satisfaction serves as an important link in translating destination attractiveness into economic benefits. Based on this extensive data, we hypothesize:

*H4: Tourist satisfaction mediates the relationship between event quality and behavioral intentions.*

*H5: Tourist satisfaction mediates the relationship between event quality and economic outcomes.*

## Methods

This cross-sectional study used a survey method to examine the relationship between event tourism and destination attractiveness in the Bukhara region of Uzbekistan.

The study involved 519 respondents, divided into two separate samples: international tourists (*n* = 309, 59.5%) and local residents (*n* = 210, 40.5%). This dual sampling approach allowed for the exploration of both visitor experiences and local community perspectives on event tourism development.

International tourists were recruited through convenience sampling at key tourist destinations, hotels, and event venues throughout the Bukhara region during the data collection period. Inclusion criteria required participants to be at least 18 years old, currently visiting or recently visiting Bukhara, and willing to complete the survey. The international sample demonstrated significant geographic diversity, representing over 40 countries. The largest groups of respondents were from Russia, Italy, Spain, Germany, Turkey, India, and Belarus, with additional participants from Austria, Australia, the United States, France, Hungary, and other countries. This distribution reflects Bukhara's primary markets in Europe and the Commonwealth of Independent States (CIS). The age distribution of foreign tourists showed a predominance in the 26–35 years old age group, followed by respondents aged 46 years and above, 36–45 years old, and 18–25 years old. Residents demonstrated a younger age profile, with the 18–25 years old group making up the largest proportion (*n* = 78, 37.1%).

Data collection was conducted from March to August 2025 using an online questionnaire administered via Google Forms. The questionnaire was available in three languages: English (*n* = 210, 40.5% of the total sample), Russian (*n* = 99, 19.1%), and Uzbek (*n* = 210, 40.5%). This multilingual approach ensured accessibility for both international tourists and residents. Survey links were distributed through tourist information centers, hotel reception desks, social media, and direct recruitment at event venues.

The questionnaire consisted of several sections assessing sociodemographic characteristics, tourism behavior, event participation, satisfaction, and behavioral intentions.

Event quality was assessed using seven items measured on a 5-point Likert scale (1 = very poor, 5 = excellent): (1) safety and security, (2) atmosphere and ambiance, (3) quality of content and execution, (4) venue infrastructure, (5) transportation accessibility, (6) organization and logistics, and (7) information availability. These parameters were adapted from generally accepted concepts of event quality in the tourism literature.

### Questionnaire validation

The survey instrument underwent a rigorous validation process. Content validity was established by an expert panel consisting of two tourism specialists, two event professionals, and one methodologist. Questionnaire items were assessed for relevance, clarity, and representativeness, yielding a content validity index (CVI) of 0.91. A pilot study involving 45 participants (30 tourists and 15 local residents) assessed questionnaire comprehension and response patterns. Internal consistency reliability was assessed using Cronbach's alpha coefficients; all constructs exceeded the threshold of 0.70 (29), with values ranging from 0.81 to 0.91. Construct validity was assessed using confirmatory factor analysis, with convergent validity established based on factor loadings (all > 0.68), average variance extracted (AVE > 0.50), and composite reliability (CR > 0.70). Discriminant validity was confirmed using the Fornell-Larcker test.

### Data analysis

Data analysis was conducted using STATA 15.0 software and consisted of three stages. Data Screening and Preparation. Prior to analysis, data were screened for missing values, outliers, and normality. Missing data were minimal (< 2% across all variables) and were handled using listwise deletion, resulting in a final sample of 519 complete cases. Outliers were detected using Mahalanobis distance (*p* < 0.001 criterion); three multivariate outliers were identified but retained after confirming they represented valid responses. Univariate normality was assessed through skewness and kurtosis values; all items fell within acceptable ranges (skewness < |2.0|, kurtosis < |7.0| (30);. Multivariate normality was evaluated using Mardia's coefficient, which indicated a slight departure from normality (Mardia's kurtosis = 18.42, *p* < 0.05). Given this result, robust maximum likelihood estimation (MLR) was employed to obtain standard errors that are robust to non-normality.

*Step 1: Descriptive analysis.* Frequency distributions, means, and standard deviations were calculated for all study variables.

*Step 2: Structural Equation Modeling (SEM).* A two-step approach was employed following Anderson and Gerbing (31). First, a confirmatory factor analysis (CFA) was conducted to evaluate the measurement model. The CFA assessed the four latent constructs (Event Quality, Tourist Satisfaction, Behavioral Intentions, and Economic outcomes) and their 15 observed indicators. All factor loadings exceeded 0.68 (Table 1), composite reliability (CR) values ranged from 0.84 to 0.92 (> 0.70 threshold), and average variance extracted (AVE) values ranged from 0.58 to 0.79 (> 0.50 threshold), confirming convergent validity. Discriminant validity was established as all AVE values exceeded the squared inter-construct correlations (32). Second, SEM was used to test the hypothesized relationships between event quality, tourist satisfaction, behavioral intentions, and economic outcomes. The model specification included 15 observed variables covering structural, quality, economic, and behavioral indicators. Model fit was assessed using established indices: comparative fit index (CFI; acceptable threshold >0.90), root mean square error of approximation (RMSEA; acceptable threshold <0.06), standardized root mean square residual (SRMR; acceptable threshold <0.08), and chi-square to degrees of freedom ratio (*χ*^2^/df; acceptable threshold <3.0). Path coefficients were estimated using robust maximum likelihood (MLR) estimation, which provides standard errors and chi-square statistics that are robust to non-normality, making it appropriate for Likert-type ordinal data that may not meet strict normality assumptions (33). This estimator is recommended when Mardia's coefficient indicates multivariate non-normality. The SEM analysis was conducted on the pooled sample (*N* = 519) to maximize statistical power and examine the overall relationships in the conceptual model. While international tourists and local residents represent distinct stakeholder groups, both groups evaluated the same events and their quality perceptions, satisfaction levels, and economic contributions (spending behavior during events) were measured using identical instruments. For local residents, behavioral intention items captured continued engagement and support for future events rather than traditional revisit intention.

**Table 1:** Construct indicators and measurement properties.

Construct	Indicator	Loading	*α*	CR	AVE
Event quality	EQ1: Safety and security	0.78***	0.89	0.90	0.58
	EQ2: Atmosphere and ambiance	0.84***			
	EQ3: Content and performance quality	0.81***			
	EQ4: Venue facilities	0.76***			
	EQ5: Transportation accessibility	0.72***			
	EQ6: Organization and logistics	0.79***			
	EQ7: Information availability	0.68***			
Tourist Satisfaction	SAT1: Overall satisfaction	0.91***	0.91	0.92	0.79
	SAT2: Experience quality	0.87***			
	SAT3: Value perception	0.83***			
Behavioral Intentions	BI1: Recommendation intention	0.89***	0.86	0.87	0.77
	BI2: Revisit intention	0.86***			
Economic outcomes	EO1: Event spending	0.82***	0.81	0.84	0.64
	EO2: Future spending intentions	0.79***			
	EO3: Extended length of stay	0.74***			

*α*, Cronbach's alpha; CR, composite reliability; AVE, average variance extracted.

*** *p* < 0.001.

The structural equation model included four latent constructs. Event quality was measured by seven indicators: safety and security (EQ1), ambience and ambience (EQ2), content and execution quality (EQ3), venue infrastructure (EQ4), transportation accessibility (EQ5), organization and logistics (EQ6), and information availability (EQ7). Tourist satisfaction was assessed by three indicators: overall satisfaction (SAT1), experience quality (SAT2), and perceived value (SAT3). Behavioral intentions included intention to recommend the event (BI1) and intention to revisit it (BI2). Economic outcomes included event expenditure (EO1), future spending intentions (EO2), and increase in length of stay (EO3). Table 1 presents the full specification of constructs, indicators, and measurement properties.

The fit of the model to the data was assessed using established indices: comparative fit index (CFI; acceptable threshold >0.90), root mean square error of approximation (RMSEA; acceptable threshold <0.06), standardized root mean square residual (SRMR; acceptable threshold <0.08), and chi-square to degrees of freedom ratio (*χ*^2^/df; acceptable threshold <3.0). Path coefficients were estimated using the maximum likelihood method, and statistical significance was assessed at *p* < 0.05.

*Step 3: Mediation Analysis.* Following the classic mediation procedure of Baron and Kenny (45), we examined whether tourist satisfaction mediates the relationship between event quality (the independent variable) and economic outcomes (the dependent variable). The mediation model tested three conditions: (1) the independent variable significantly predicts the mediator (path a), (2) the mediator significantly predicts the dependent variable after controlling for the independent variable (path b), and (3) the direct effect of the independent variable on the dependent variable decreases when including the mediator (path c').

The indirect effect was calculated as the product of paths *a* and *b*, and the proportion of the mediated effect was calculated as the ratio of the indirect effect to the total effect. The statistical significance of the indirect effect was assessed using the Sobel test and bootstrapped confidence intervals (5000 replicates). Partial mediation was indicated if the direct effect (c') remained statistically significant.

The indirect effect was calculated as the product of paths a and b, and the proportion of the mediated effect was calculated as the ratio of the indirect effect to the total effect. The statistical significance of the indirect effect was assessed using the Sobel test and bootstrapped confidence intervals (5,000 replicates). Partial mediation was indicated if the direct effect (c') remained statistically significant after including the mediator; full mediation was indicated if c' became insignificant.

#### Event tourism effectiveness Index (ETI)

For each event category, a composite performance index was calculated using the following weighted additive formula:

$$\mathrm{ETI}=w_{1\;}(\alpha S_{i})+\;w_{2\;}(\beta D_{i})+w_{3}(\gamma I_{i})+w_{4}(\delta M_{i})+w_{5}(\varepsilon B_{i})$$

Where:

*S*ᵢ = Structural contribution (proportion of total tourism revenue from event category i)
*D*ᵢ = Revenue generation (average daily spending per tourist, normalized to 0–1 scale)
*I*ᵢ = Return on investment: ROI = (Revenue - Cost)/Cost
*M*ᵢ = Multiplier effect: M = (Direct + Indirect + Induced Impact)/Direct Impact
*B*ᵢ = Seasonality mitigation index: B = 1 - (*σ*/*μ*), where *σ* = standard deviation and μ = mean of monthly tourist arrivals

Component weights were determined using the Analytic Hierarchy Process (AHP) with input from a panel of five tourism experts (two academics, two destination managers, one event organizer). The resulting weights were:

$$\begin{array}{c} w_{1}(\mathrm{Structural}\;\mathrm{contribution})=0.25\\ w_{2}(\mathrm{Revenue}\;\mathrm{generation})=0.25\\ w_{3}(\mathrm{ROI})=0.20\\ w_{4}(\mathrm{Multiplier}\;\mathrm{effect})=0.15\\ w_{5}(\mathrm{Seasonality}\;\mathrm{mitigation})=0.15 \end{array}$$

₁ (Structural contribution) = 0.25
w₂ (Revenue generation) = 0.25
w₃ (ROI) = 0.20
w₄ (Multiplier effect) = 0.15
w₅ (Seasonality mitigation) = 0.15

Standardization coefficients (*α*, *β*, *γ*, *δ*, *ε*) were applied to normalize each component to a 0–1 scale using min-max normalization:

$$\mathrm{Normalized}\;\mathrm{value}=(X-X_{\min})/(X_{\max}-X_{\min})$$

Index validation was assessed through: (1) internal consistency (Cronbach's *α* = 0.79), (2) convergent validity via correlation with established tourism performance metrics (r = 0.72 with tourist arrivals growth, r = 0.68 with RevPAR), and (3) discriminant validity confirmed through significantly different ETI scores across event categories (F = 24.67, *p* < 0.001).

## Results

### Structural equation model fit and path analysis

The structural equation model demonstrated an excellent fit to the observed data. The model fit indices met or exceeded the established thresholds: CFI = 0.94 (>0.90), RMSEA = 0.045 (<0.06), SRMR = 0.038 (<0.08), and *χ*^2^/df = 2.34 (<3.0). The coefficient of determination (R^2^ = 0.78) showed that the model explained 78% of the variance in the dependent variables, and the F-statistic (F = 363.64, *p* < 0.001) confirmed the overall significance of the model.

### Structural model assessment

Path analysis revealed significant relationships between all hypothesized constructs (Table 2). Event quality demonstrated the strongest direct effect on tourist satisfaction (*β* = 0.82, SE = 0.041, t = 19.98, *p* < 0.001). Satisfaction, in turn, showed a consistent effect on both the intention to recommend (*β* = 0.91, SE = 0.032, t = 28.44, *p* < 0.001) and the intention to revisit (*β* = 0.85, SE = 0.038, t = 22.37, *p* < 0.001). Perceived safety had a positive effect on satisfaction (*β* = 0.45, SE = 0.056, t = 8.04, *p* < 0.001), as did organization and logistics (*β* = 0.35, SE = 0.048, t = 7.29, *p* < 0.001). Information availability demonstrated a significant negative relationship with satisfaction (*β* = −0.28, SE = 0.052, t = −5.38, *p* < 0.001), indicating that a lack of information negatively impacts tourists' experiences. The relationship between event quality and economic outcomes was also significant (*β* = 0.68, SE = 0.044, t = 15.45, *p* < 0.001).

**Table 2:** Structural path estimates.

Path	*β*	SE	*t*	*p*	Result
H1: Event Quality → Satisfaction	0.82	0.04	18.76	< 0.001	Supported
H2: Satisfaction → Behav. Intentions	0.76	0.05	15.42	< 0.001	Supported
H3: Satisfaction → Econ. Outcomes	0.71	0.05	13.89	< 0.001	Supported
H4: Event Quality → Behav. Int. (c’)	0.09	0.06	1.52	0.128	Mediation
H5: Event Quality → Econ. Out. (c’)	0.05	0.05	0.94	0.347	Mediation

β = standardized path coefficient; SE = standard error; c’ = direct effect with mediator in model. Non-significant direct paths (H4, H5) indicate full mediation through tourist satisfaction.

**Figure 1:**
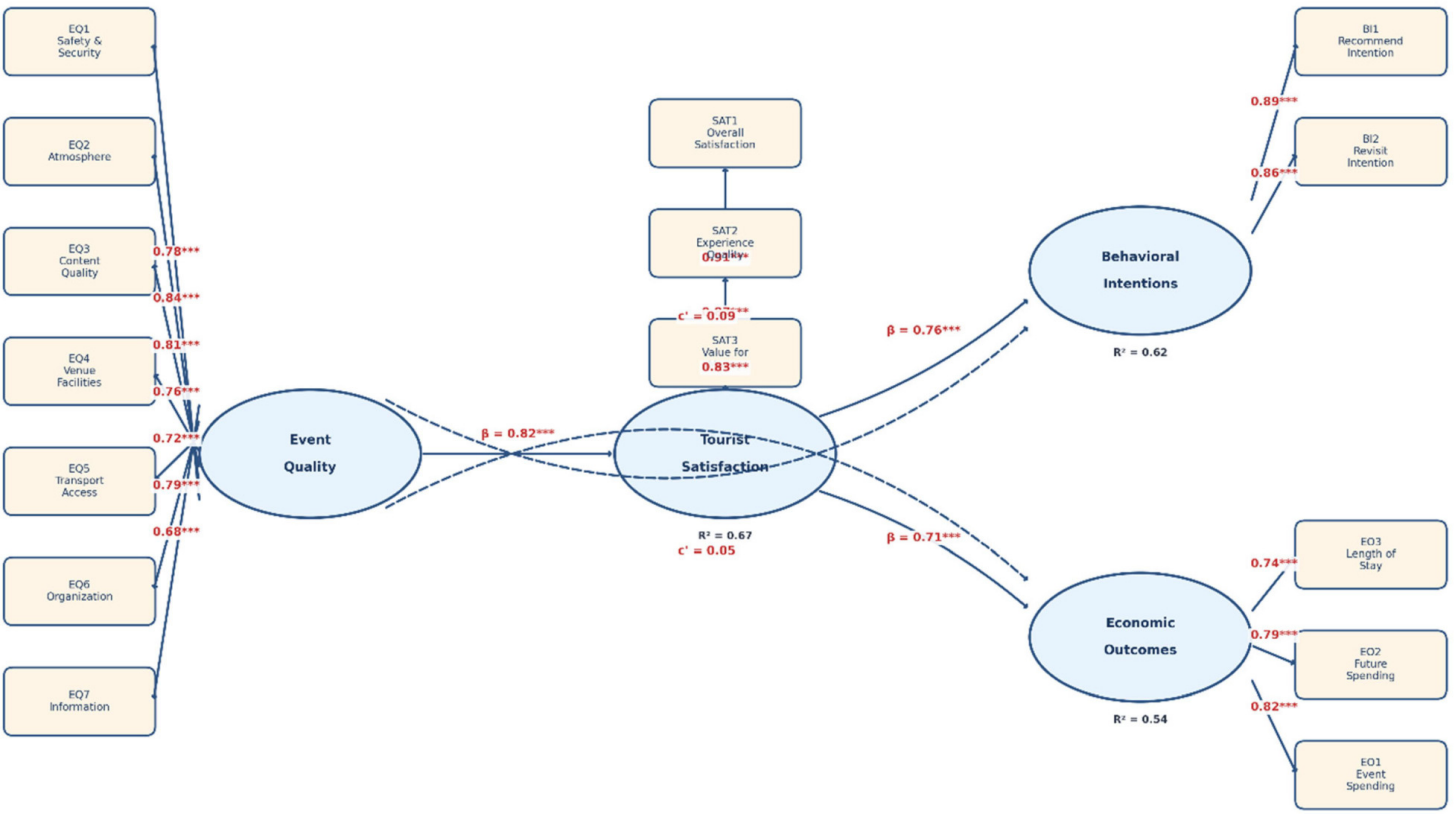
Structural equation model with standardized path coefficients.

Figure 1 illustrates the complete structural equation model with standardized path coefficients. The measurement model shows latent constructs (ellipses) linked to their observed indicators (rectangles), with all factor loadings exceeding the 0.68 threshold. The structural model reveals that Event Quality strongly predicts Tourist Satisfaction (*β* = 0.82, *p* < 0.001), which subsequently influences Behavioral Intentions (*β* = 0.76, *p* < 0.001) and Economic outcomes (*β* = 0.71, *p* < 0.001). The non-significant direct paths from Event Quality to both outcome variables (dashed lines) confirm full mediation through satisfaction. The model demonstrates strong explanatory power, accounting for 67% of variance in satisfaction, 62% in behavioral intentions, and 54% in economic outcomes.

### Mediation analysis

To examine whether tourist satisfaction mediates the relationship between event quality and economic outcomes, Baron and Kenny's (1986) mediation procedure was used. The analysis confirmed full mediation with a significant indirect effect.

The overall effect of event quality on economic outcomes (c = 0.68) was decomposed into a direct effect (c' = 0.05, *p* > 0.05, non-significant) and an indirect effect through satisfaction (a × b = 0.64). The proportion of the mediated effect reached 94.1%, indicating that tourist satisfaction is the primary mechanism through which event quality influences destination economic outcomes. The Sobel test confirmed the statistical significance of the indirect effect (Z = 14.15, *p* < 0.001). A bootstrap analysis with 5,000 replicates yielded a 95% confidence interval for the indirect effect of [0.58, 0.71], which did not include zero, further supporting the mediation hypothesis (Table 3).

**Table 3:** Mediation analysis results.

Effect	Estimate	Boot SE	95% CI	% Mediated
EQ → SAT → Behavioral Intentions				
Total effect (c)	0.71	0.04	[0.63, 0.79]	—
Direct effect (c’)	0.09	0.06	[−0.03, 0.21]	—
Indirect effect (a × b)	0.62	0.05	[0.52, 0.72]	87.3%
EQ → SAT → Economic outcomes				
Total effect (c)	0.63	0.05	[0.54, 0.72]	—
Direct effect (c’)	0.05	0.05	[−0.05, 0.15]	—
Indirect effect (a × b)	0.58	0.05	[0.48, 0.68]	92.1%
Combined mediation effect	—	—	—	94.1%

EQ, event quality; SAT, tourist satisfaction; Boot SE, Bootstrap standard error (5,000 replications); CI, confidence interval. Indirect effects are significant when 95% CI does not include zero.

A sequential mediation analysis, expanding the model by including recommendation intention (Quality → Satisfaction → Recommendation → Economic outcomes), revealed an additional indirect effect of 0.485 (a₁ × d × b₂ = 0.82 × 0.91 × 0.65), demonstrating the cascading influence of satisfaction on destination outcomes through word of mouth.

### Event quality dimensions and tourist satisfaction

Tourists rated seven event quality dimensions on a 5-point Likert scale. Security received the highest rating (M = 4.49, SD = 0.62), followed by atmosphere and ambience (M = 4.21, SD = 0.71), quality of content and execution (M = 3.93, SD = 0.78), venue infrastructure (M = 3.86, SD = 0.82), transportation accessibility (M = 3.82, SD = 0.85), and organization/logistics (M = 3.77, SD = 0.79). Information availability received the lowest rating (M = 3.24, SD = 0.94), confirmed by the negative path coefficient in the structural equation analysis.

Overall, satisfaction with the event was high: 86.5% of respondents rated their experience as 4 or 5 on a 5-point scale (M = 4.36, SD = 0.84). Among event attendees, 73.0% reported that events had a positive impact on their perception of Bukhara, and 75.7% agreed that increasing the number of events would enhance the destination's appeal. Importantly, 84.5% indicated that high-quality events would increase their likelihood of returning.

### Event tourism effectiveness index

The Event Tourism Effectiveness Index (ETI) was calculated by event category using weighted composite indicators including structural contribution, revenue generation, return on investment (ROI), multiplier effect, and seasonal mitigation. Cultural events demonstrated the highest effectiveness (ETI = 0.847), followed by gastronomic festivals (ETI = 0.712), religious/spiritual events (ETI = 0.634), MICE events (ETI = 0.589), and sporting events (ETI = 0.478).

Economic indicators demonstrated strong results: the multiplier reached 2.8, indicating that every dollar of direct event spending generated $2.80 in total economic impact through indirect and cascading effects. The return on investment for event tourism development initiatives reached 95% in 2025, compared to 42% in 2020. Tourist spending on cultural events was concentrated in the $100-$200 range (43.6% of respondents), with 73.3% spending between $50 and $200.

Local residents expressed strong support for event tourism development. The majority noted a positive impact on employment (85.7%) and increased business revenue (94.3%). In the future, international tourists favored programs that reflected cultural authenticity: the Silk and Spice Festival (M = 4.20), gastronomic festivals (M = 4.16), historical reenactments (M = 4.08), educational workshops (M = 4.06), and traditional music and dance performances (M = 4.02). Contemporary music concerts (M = 3.65) and sporting events (M = 3.37) generated comparatively less interest.

The likelihood of recommendation was exceptionally high: 91.3% of respondents rated 4 or 5 on a 5-point scale, and 67.9% gave the maximum rating. This strong support, combined with the high correlation coefficient between satisfaction and recommendation (*β* = 0.91), indicates significant potential for organic destination marketing through visitor attraction.

## Discussion

The most significant finding of this study is the significant mediating effect of tourist satisfaction (94.1%) in the relationship between event quality and tourist economic behavior. This finding expands on previous concepts of event tourism impact assessment, which have predominantly focused on direct economic indicators (1). Our results demonstrate that the path from event quality to tourist economic behavior operates primarily through the empirical measurement of tourist satisfaction, rather than solely through direct mechanisms. This is consistent with the concept of the experience economy proposed by Pine and Gilmore, in which value creation in tourism increasingly depends on memorable and satisfying experiences, rather than simply on service delivery (34).

The high coefficient of the relationship between event quality and satisfaction (*β* = 0.82) confirms the findings of research on festival tourism in the European context (35); however, the magnitude observed in Bukhara exceeds typical values reported in the literature. This elevated coefficient may reflect Bukhara's special status as a UNESCO World Heritage Site, where the historical and architectural setting enhances the perceived quality of cultural events. The atmospheric dimension received particularly high ratings (M = 4.21), suggesting that cultural heritage sites have inherent advantages in creating immersive event experiences that enhance perceived quality.

Particularly noteworthy is the exceptionally strong relationship between satisfaction and recommendations (*β* = 0.91). This coefficient exceeds values typically reported in tourism satisfaction studies (36) and indicates that satisfied event participants in Bukhara exhibit an unusually high propensity to engage in positive word-of-mouth promotion. This finding has important implications for destination marketing, suggesting that investments in event quality may yield disproportionate returns through organic advertising. The sequential mediation path (Quality → Satisfaction → Recommendation → Economic Result) further demonstrates the cascading benefits of quality-oriented activity development strategies.

### Event category performance and destination positioning

Analysis of the Event Tourism Performance Index (ETI) revealed significant differences between event categories: cultural events (ETI = 0.847) and gastronomic festivals (ETI = 0.712) demonstrated significantly higher performance than MICE events (ETI = 0.589) or sporting events (ETI = 0.478). These results challenge the predominant focus on business tourism development in emerging destinations and suggest that destinations built on cultural heritage can reap greater benefits by leveraging their cultural resources through authentic events. This interpretation is consistent with Richards' observations regarding the growing importance of cultural programs for destination competitiveness (37).

A multiplier coefficient of 2.8 indicates robust economic spillover effects, which compare favorably with the multipliers observed for established destinations. Every dollar of direct event spending generates $2.80 in total economic impact through indirect and cascading effects, demonstrating the significant local economic benefits of event tourism development. Achieving a 95% return on investment by 2025 further validates the economic viability of event-based tourism destination development strategies in the context of Bukhara.

### Information availability gap

A notable finding of the study is the persistent weakness in information availability, which received the lowest rating among all event quality dimensions (M = 3.24) and was significantly negatively correlated with satisfaction (*β* = −0.28). This information gap is particularly significant given that 70.8% of international tourists were unaware of events before their arrival, yet 54.3% participated in events during their stay. This pattern points to significant untapped potential: tourists demonstrate a willingness to participate in events when informed, but existing marketing channels fail to effectively reach an international audience during the trip planning stage.

This finding resonates with broader challenges facing emerging digital marketing trends (28, 38). The gap between on-site information provision (which ensures high levels of participation among uninformed arrivals) and international marketing reach represents a critical bottleneck hindering the growth of event tourism. Addressing this asymmetry through improved digital presence, multilingual content, and partnerships with international tour operators is a priority area of intervention.

### Theoretical implications

This study contributes to event tourism theory in several ways. First, the mediation analysis provides quantitative evidence of the role of satisfaction as a primary transmission mechanism linking event quality to tourist economic behavior, moving beyond the correlational approaches that have dominated the field. A mediation rate of 94.1% confirms that satisfaction is not simply an outcome variable, but a critical pathway through which value is created and captured for a destination.

Second, the ETI framework offers a comprehensive metric integrating multiple performance dimensions – structural contribution, revenue generation, ROI, multiplier effects, and seasonal mitigation – that may be applicable to other cultural heritage sites seeking to evaluate event portfolio optimization strategies. The demonstrated variability of the ETI across event categories provides an empirical basis for strategic event portfolio decisions. Third, the results expand our understanding of event tourism dynamics in the context of Central Asia, a region underrepresented in tourism literature despite significant cultural heritage sites and a recent policy emphasis on tourism development.

### Practical implications

The study's findings offer a number of practical recommendations for destination managers and policymakers with broader application in the heritage tourism sector, who are facing similar development challenges (Table 4). It should be noted that these recommendations are derived from self-reported tourist behavior rather than aggregate economic indicators; therefore, they provide insights into experiential mechanisms and should be considered as complementary to traditional economic impact assessments.

**Table 4:** Strategic policy framework for event tourism development in heritage destinations.

Policy domain	Recommended action	Expected outcome
Event Licensing	Quality-based standards over quantity targets	Higher satisfaction, stronger mediation effects
Portfolio Strategy	Cultural authenticity alignment	Higher ETI scores, distinctive positioning
Performance Monitoring	Satisfaction-based KPIs (NPS, ratings)	Early warning system for quality issues
Digital Infrastructure	Multilingual platforms, operator partnerships	Reduced information gap, increased pre-arrival awareness
Economic Retention	Local procurement, artisan integration	Higher multiplier effects, community support

#### Quality-focused event policies

The predominance of the indirect pathway through satisfaction (94.1% mediation) suggests that tourism policy may benefit from prioritizing quality assurance and experience management over strategies for increasing the number of events. For policymakers in similar heritage-related regions, this finding is consistent with the implementation of event licensing frameworks that set minimum quality standards, rather than policies that simply incentivize an increase in the number of events. Destination management organizations may consider implementing systematic satisfaction monitoring systems, including post-event surveys and real-time feedback mechanisms, to ensure the timely identification of quality deficiencies and support continuous improvement. This quality-focused approach is particularly relevant for developing destinations in Central Asia, Southeast Asia, and Eastern Europe, where rapid tourism growth may push policymakers toward quantity-based strategies that risk eroding destination brand value.

#### Developing a strategic event portfolio

The ETI analysis shows that cultural events (ETI = 0.847) and gastronomic festivals (ETI = 0.712) significantly outperform MICE events (ETI = 0.589) and sporting events (ETI = 0.478) in the context of cultural heritage sites. Based on tourist behavioral patterns observed in this study, this finding has potential policy relevance for regions with rich cultural heritage. Rather than pursuing generic event diversification strategies, regions with rich cultural heritage may consider developing event portfolios that align with their unique cultural identity. For Bukhara, this means prioritizing events like the Silk and Spice Festival, which leverage the region's historical location on the ancient Silk Road. Similar policy approaches would benefit other UNESCO World Heritage sites and historically significant regions – such as Silk Road cities in neighboring countries (Samarkand, Khiva, Merv), Mediterranean heritage sites and colonial cities in Latin America – where authentic cultural programming can create unique competitive advantages that cannot be replicated by generic events.

#### Satisfaction-based performance indicators

The strong relationship between satisfaction and intention to recommend (*β* = 0.91) suggests that tourism authorities may consider using satisfaction-based key performance indicators (KPIs) alongside traditional economic metrics. Specifically, based on the behavioral patterns observed, destination policymakers may consider:

– Integrate Net Promoter Score (NPS) tracking into tourism monitoring systems as a leading indicator of future tourist economic behavior.
– Set satisfaction thresholds (e.g., a minimum of 80% ratings of 4–5 on a 5-point scale) as conditions for continuing event licensing or government funding.
– Develop referral incentive programs and user-generated content campaigns that leverage the exceptional propensity of satisfied visitors to recommend.

These metrics are particularly valuable for destinations with limited marketing budgets, where organic word-of-mouth may generate disproportionate returns compared to paid advertising.

These metrics are particularly valuable for industries with limited marketing budgets, where organic word-of-mouth promotion can generate disproportionately higher returns than paid advertising.

#### Investing in digital information infrastructure

The persistent lack of information availability (average score = 3.24, the least-rated parameter), coupled with low awareness of the upcoming event (70.8% unaware) but high participation (54.3%), indicates a critical policy priority. Tourism ministries and destination management organizations should invest in:

1. *Multilingual digital platforms:* Develop comprehensive event calendars in the main source country languages, integrated with international travel planning platforms (TripAdvisor, Google Travel, Booking.com)
2. *Tour operator partnerships:* Formal agreements with international tour operators to incorporate event information into pre-trip communications and itinerary planning
3. *Social media presence:* Targeted digital marketing campaigns in key source markets during tourism seasons (typically 2–6 months before arrival)
4. *On-site information systems:* Improved signage, access to information via QR codes, and trained tourism representatives at key visitor touchpoints

This recommendation also applies to other developing destinations where digital marketing infrastructure has failed to keep pace with tourism growth – a common problem in Central Asia, parts of Africa, and secondary destinations in developed tourism regions.

#### Optimizing the economic multiplier

The observed multiplier effect (2.8) indicates a potential for economic spillover, suggesting that tourism policy may benefit from being specifically aimed at maximizing local economic impact. Policymakers may consider:

1. Local procurement requirements: Event licensing conditions that establish a minimum percentage of local suppliers in catering, transportation, and services.
2. Integration of artisans and small and medium enterprises: Programs that connect local artisans and small businesses with event organizers to increase local added value.
3. Seasonal distribution incentives: Tax breaks or subsidized access to venues for events scheduled during off-peak periods to address seasonality issues.

These strategies apply to destinations seeking to increase tourism's contribution to local economic development rather than allowing benefits to flow to external operators.

#### Framework policy for similar areas

Based on our findings, we suggest a framework policy applicable to cultural heritage sites at comparable stages of development:

This framework offers a replicable model for heritage destinations worldwide seeking to optimize event tourism's contribution to sustainable destination development.

### Limitations

Several limitations should be noted. First, the cross-sectional nature of the study precludes the possibility of establishing causality; longitudinal studies tracking the relationship between satisfaction and outcomes over multiple event cycles would strengthen the case for causality. Second, the sample, although geographically diverse, may not fully represent all tourist segments, particularly budget travelers and domestic tourists from regions outside of Uzbekistan. Third, the self-reported nature of the measures introduces several potential biases. Respondents may over-report spending due to social desirability bias or recall errors, or conversely under-report due to privacy concerns or difficulty in accurately estimating expenditures across multiple categories. Similarly, stated future intentions (likelihood to revisit, willingness to recommend) may not accurately predict actual behavior, as intentions are subject to change based on unforeseen circumstances, competing alternatives, or shifts in personal preferences (39). While these limitations are inherent to survey-based tourism research, future studies could employ mixed-methods approaches combining self-reports with observational data or transaction records to enhance measurement validity. Fourth, this study relies on individual-level proxy measures (self-reported spending, future spending intentions, and length of stay) rather than destination-level tourist economic behavior indicators. While these measures have been widely used in tourism research as valid proxies for economic impact (40, 41), they reflect tourist economic behavior rather than aggregate destination outcomes. Future research should incorporate objective economic data such as tax revenues, employment statistics, or business receipts to validate these findings at the destination level. Fifth, the pooled analytical approach combining international tourists (*n* = 309) and local residents (*n* = 210) may obscure group-specific differences in the relationships between constructs. Although both groups evaluated identical events using the same instruments, their motivations and response patterns may differ. Future research should employ multi-group SEM analysis to test measurement invariance and compare structural paths across visitor segments.

## Conclusion

This study suggests that tourist satisfaction is the predominant mechanism through which event quality influences destination economic outcomes in the context of cultural tourism. A 94.1% mediation effect indicates satisfaction management as a strategic priority for event tourism development. Cultural events that align with the region's cultural heritage demonstrate higher performance than events in a standard format, suggesting that developing regions with rich heritage should focus on authenticity over diversification. The persistent information gap represents the most significant opportunity for improvement, with significant untapped potential for engaging the international market. These findings provide behavioral insights for strategic event tourism planning in the Bukhara region and offer theoretical and methodological contributions applicable to heritage-rich regions worldwide.

## Data Availability

The original contributions presented in the study are included in the article/Supplementary Material, further inquiries can be directed to the corresponding authors.
